# Treatment of multicentric Castleman's Disease accompanying multiple myeloma with bortezomib: a case report

**DOI:** 10.1186/1756-8722-2-19

**Published:** 2009-04-28

**Authors:** Zhen-gang Yuan, Xiao-yi Dun, Yong-hua Li, Jian Hou

**Affiliations:** 1 Department of Hematology, Second Affiliated Hospital to the Second Military Medical University, 415 Fengyang Rd, Shanghai 200003, PR China

## Abstract

Multicentric Castleman's disease (MCD) is a rare lymphoproliferative disorder of unknown etiology and characterized by various clinical manifestations and multiple organ involvement. It has been reported in association with POEMS syndrome and can progress to Kaposi's sarcoma or malignant lymphoma. The disease runs a more aggressive course and a poor prognosis. Optimal therapies have not been well established up to now. We here reported a case of rare MCD complicated with multiple myeloma who received bortezomib and achieved very good remission. To our knowledge, this is the first report on MCD in the setting of multiple myeloma with good response to bortezomib.

## Background

Multicentric Castleman's disease (MCD) was first described as an entity in 1978 by Gaba et al[1]. The clinical manifestations of MCD are heterogeneous and usually with multiple organ involvement. It has been reported in association with POEMS syndrome (but never with MM up to now) and can progress to Kaposi's sarcoma or malignant lymphoma [2]. Treatment for MCD remains suboptimal. Bortezomib is a novel proteasome inhibitor that affects myeloma cell growth by NF-κB blockade [3]. Clinical trials have clearly demonstrated that bortezomib is active in patients with relapsed and refractory MM. To explore the efficacy of bortezomib in MCD therapy, we successfully treated a 70-year-old male patient who had both MCD and multiple myeloma with bortezomib, and a good remission was observed.

## Case presentation

A 70-year-old male patient was admitted to our hospital in May 2007 with chief complaints of left upper abdomen distention for 1 year with progressive peripheral lymphadenopathy associated with 8 kg weigh loss over 7 months. The patient had epigastric discomfort with no fever and night sweat initially in May 2006. Abdominal computed tomographic (CT) scan and ultrasonography revealed splenomegaly and many enlarged retroperitoneal lymph nodes. Bone marrow cytomorphologic examination and biopsy at that time were normal. Subsequently, the distention increased gradually in severity and icteric sclera was seen. Five months later, painless and slowly-enlarging bilateral latero-cervical lymphadenopathy had developed. He also had episodes with petechia throughout the whole body accompanied with fatigue, low-grade fever and night-sweat in the last month. He never had numbness/tingling in his limbs during the course of the illness.

Physical examination revealed a chronically-ill appearance with enlarged lymph nodes in the cervical, supraclavicular, axillar, and inguinal regions, the biggest of which was 3.5 cm × 2.5 cm in size. Splenomegaly (10 cm below the left costal margin)with no hepatomegaly was also palpated.

Blood counts showed mild anemia (hemoglobin, 99 g/L), a white blood cell count of 2.3 × 10^9^/L and a low platelet count of 30 × 10^9^/L. Serum protein was 112 g/L (albumin 23 g/L, globulin 89 g/L). Immunoelectrophoresis showed monoclonal increase in serum immunoglobulin with IgG-κ. The serum IgG, IgA, IgM, κ and λ values were 86.8, 0.73, 0.63, 26.3 and 10.5 g/L, respectively. The total Bence-Jones protein in 24-hour urine was 1850 mg. The serum β2-microglobulin level was 8.09 mg/L and CRP level was 16.6 mg/L. Anti-nuclear antibodies and serologic tests (Epstein-Barr virus, hepatitis B and C viruses, cytomegalovirus and human immunodeficiency virus) were negative. All of the serum tumor markers(CEA, AFP, CA125, CA19-9, PSA, NSE) were negative. The thyroid function tests of T3, T4 and TSH were normal.

**Figure 1:**
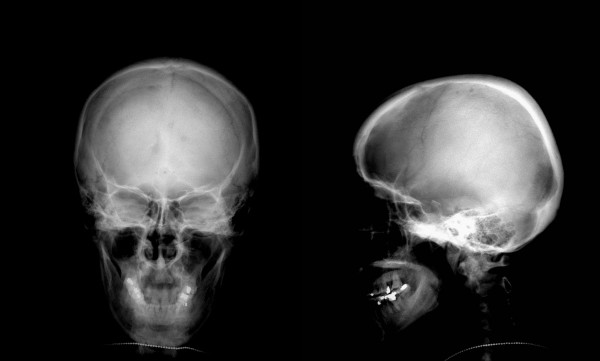
**X-ray revealed low density foci on the skull(posterior-anterior and lateral film)**.

**Figure 2:**
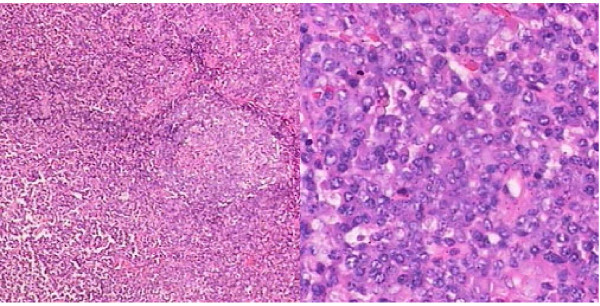
**Pathology of an enlarged cervical lymph node was compatible with Castleman's disease, plasma cell type (H & E stain: 400× and 1000×)**.

**Figure 3:**
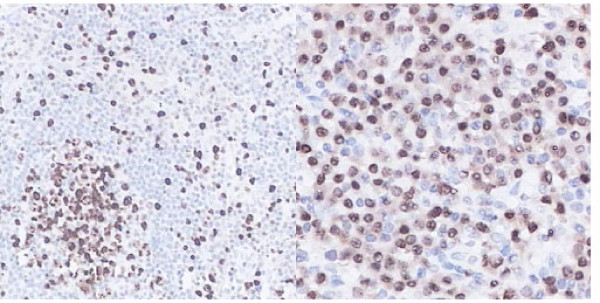
**Immunohistochemistry showed Vs38(+) (400× and 1000×)**.

Bone marrow cytomorphologic examination at this hospitalization showed increased plasma cells at 11%. Bone X-ray revealed low density foci on the skull (Figure 1). Electromyelogram showed normal nerve conduction velocity of cubital nerve and median nerve. Ultrasound examination of the abdomen revealed marked splenomegaly with 200 mm × 90 mm in size. Abdominal CT scan confirmed splenomegaly and enlarged lymph nodes in retroperitoneal regions. Biopsy of a cervical lymph node revealed that the structure of lymph node was still existed and most of the folliculus lymphaticus were infiltrated with sheets of plasma cells both in the germinal centre and in the interfollicular space, and absence of vascular proliferation (Figure 2). Immunohistochemistry for the mature plasma cell in the germinal centers showed LCA(+), CD20(±), CD79(±), CD15(-), CD30(±), CD7(±), MUM1(+), Vs38(+), Ki67(++), and polyclonal κ as well as λ was positive (Figure 3). HHV-8 DNA was not detected by nested PCR in the paraffin embedded tissue specimens. The pathological diagnosis was plasma cell variant of Castleman's disease. With all these findings, the patient was diagnosed to have MCD complicated with multiple myeloma (Durie-Salmon IIIA). After written informed consent, the patient was given bortezomib (1.3 mg/m^2^) as an intravenous bolus twice weekly for 2 weeks on days 1, 4, 8, and 11 in a 3–4 weeks cycle. He got partial remission after two cycles. The serum IgG level, β2-microglobulin and CRP level decrease to 45.2 g/L, 5.8 mg/L and 11.2 mg/L, respectively. A 75% decrease in lymph nodes and splenomegaly was noted. In order to increase the efficacy, dexamethasone at a dose of 30 mg/day on days 1–4, 8–11 was given in combination with bortezomib at 3^rd ^and 4^th ^cycle. The treatment was stopped after a total of 4 cycles. Eighteen months after diagnosis, the patient was in very good partial remission with no lymphadenopathy. The serum IgG level was 23 g/L, β2-microglobulin was 2.1 mg/L and CRP was 3.0 mg/L.

## Discussion

MCD is more common in elder male (male/female is 2.5~13:1). It is generally of the plasma cell type or mixed variant. MCD is a systemic disease with significant peripheral lymphadenopathy and hepatosplenomegaly, as well as frequent fever, night sweats, fatigue and weight loss. Abnormal laboratory findings include pancytopenia, abnormal function of liver and kidney, raised CRP, IL-6 and hypergammaglobulinemia. However, monoclonal gammaglobulinemia is rare. In addition, it has been reported in association with amyloidosis, nephrotic syndrome, Sjögren syndrome and POEMS syndrome and can progress to Kaposi's sarcoma or malignant lymphoma.

The patient in this case was manifested with progressing splenomegaly at first and followed with enlarged peripheral lymph nodes and general symptoms such as fatigue and weight loss. Immunohistochemistry and pathology of cervical lymph node revealed the diagnosis of MCD. In addition, a monoclonal globulin spike of 86.8 g/L was found on serum electrophoresis at visit with further identification as IgG and κ light chain by immunofixation analysis. Marrow plasmacytosis of 11% plasma cells with morphological abnormality were also seen. X-ray demonstrated lytic bone lesions in the skull. So this patient was diagnosed to have multiple myeloma with one major and two minor WHO criteria. To our knowledge, there has been no report of MCD together with multiple myeloma. In 1996, Komatsu et al[4] reported a case with cervical UCD complicated with benign monoclonal gammopathy, which was presumed to be associated with increased IL-6 or the primary manifestation of multiple myeloma. Monoclonal gammopathy had occurred in Castleman's disease with POEMS syndrome. However, no sign of POEMS was found in this case, such as polyneuropathy, endocrinopathy, skin changes and edema. He has no autoimmune disorders, primary immune deficiency, HIV infection and chronic nephropaty. We presumed that the monoclonal gammopathy was probably one of the progressing features of MCD.

We could not measure serum IL-6 level due to the limitation of our lab technology, nevertheless, according to the increased level of CRP, the patient was inferred to have a high level of IL-6 which has positive correlation with CRP. In recent 10 years, IL-6 has been implicated in the pathophysiology of MCD[5]. The role of IL-6 in genesis of myeloma is demonstrated. It causes B-cell proliferation resulting in hyperplastic follicles and hence the enlarged lymph nodes. IL-6 also induces an acute phase reaction comprising increases in ESR, CRP and serum fibrinogen. B-symptom is virtually always associated with increased IL-6 levels. Dysregulated overproduction of IL-6 from germinal center B cells is implicated in the pathogenesis of plasma-cell-type Castleman's disease.

Although there is evidence that HHV-8 plays a significant role in the pathogenesis of HIV-associated MCD. Definitively establishing the causality of HHV-8 in the aetiology of other MCD type will prove challenging. Our patient has no evidence of HHV-8 infection, so we propose that the two phenomenons are linked through mechanisms involving IL-6, which could lead to future treatment options.

The MCD plasma cell subtype runs a more aggressive course with poor prognosis, and optimal therapies have not been well established. A number of therapies have been used for multicentric disease, including steroid-monotherapy and combined chemotherapy [6,7]. Other therapies include interferon-α[8], antiviral medications[9], anti-IL-6 monoclonal antibody[10] and humanized anti-human IL-6 receptor mono-clonal antibody (MRA)[11]. All-trans retinoic acid[12], thalidomide[13] and Rituximab [14-17] also have been reported. Most of these reports included only a small number of patients.

Bortezomib has recently been shown to produce significant responses in about one-third of patients with refractory and relapsed MM[18]. The mechanism of action of bortezomib is thought in part to be due to selective inhibition of the proteasome. This drug has been reported to affect myeloma cell growth by NF-κB blockade, down regulation of cytokines such as IL-6[19]. IL-6 has been implicated in the pathophysiology of MCD, as mentioned above, providing a rationale for treatment of MCD with bortezomib. Previous clinical trials have indicated that the combination of bortezomib and dexamethasone may be additive or possibly synergistic. After two cycle's treatment in the current case, the enlarged lymph nodes disappeared and splenomegaly decreased significantly. Bone marrow cytomorphologic examination showed only 3% plasma cell by ratio with normal morphology. It is noteworthy that the level of CRP which has positive correlation with IL-6 level decreased to normal range. It is likely that the effect of bortezomib on MCD is due to inhibition of IL-6 secretion.

At the 18-month follow-up, the patient showed persistent clinical improvement, with no B symptoms, weight gain, disappearance of lymphadenopathy and improvement of performance status. Therefore, this observation showed that the combination of bortezomib and dexamethasone has activity in MCD.

## Conclusion

In summary, we reported the first case of MCD with multiple myeloma which had remarkable response to the combination of bortezomib and dexamethasone. This observation showed that the combination of bortezomib and dexamethasone has activity in MCD.

## Abbreviations

MCD: Multicentric Castleman's disease; UCD: Unicentric Castleman's Disease; MM: multiple myeloma; CRP: C-Reactive Protein.

## Competing interests

The authors declare that they have no competing interests.

## Authors' contributions

All authors were involved in preparation of this manuscript, including data collection and preparation of figures.

## Consent

The patient has provided informed consent for the publication of this case report and accompanying images.
